# Is the Treatment Response Different in Treatment-naive HIV-infected Patients with Very High Viral Load (>1 Million Copies)? Three-year Data

**DOI:** 10.4274/balkanmedj.galenos.2020.2019.11.70

**Published:** 2020-08-11

**Authors:** Özlem Kandemir, Elif Şahin Horosan, Serhat Şahinoğlu

**Affiliations:** 1 Department of Infectious Diseases and Clinical Microbiology Mersin University School of Medicine, Mersin, Turkey

To the Editor,

The main objectives of antiretroviral therapy (ART) are to suppress virus replication, preserve and improve immunological functions, and reduce human immunodeficiency virus (HIV)-related morbidity and mortality. In phase 3 ART-related studies, results of patients with baseline viral load of >100,000 c/mL were reported (1,2,3), whereas data with ≥1 million c/mL in real-life study are limited. Here, we represented virological and immunological responses in patients with HIV-1 infection who had a virological load of >1 million copies and received ART with an integrase inhibitor (INI) combination.

We evaluated a single-center, retrospective study of HIV-infected adult patients who had a virological load of >1 million copies followed in our Clinic of Infectious Diseases, between 2015 and 2018. HIV-RNA value <50 c/mL at week 24 was defined as virological success. The increase in the number of CD + T lymphocyte count 50-100 cells/mm^3^ in the first year is considered as an immunological success.

A total of 16 patients were enrolled. The mean age of patients was 40 (range of 18-73) years; 93.8% of the patients were men. Eleven patients were on a single tablet regimen; 5 on elvitegravir (EVG), cobicistat, emtricitabine (FTC), and tenofovir alafenamide; 3 on EVG, c, FTC, and tenofovir disoproxil fumarate; 3 on dolutegravir, abacavir, and lamivudine; and 5 on multi-tablet therapy. The mean viral load of the patients was 4,467,618 (1,025,032-10,000,000) c/mL. The virological response of the patients to the treatment at 4^th^, 24^th^, and 48^th^ weeks are shown in Figure 1. The immunological response was detected in 87.5% (14 patients) at 24^th^ and 94% (15 patients) at 48^th^ week. None of the seven responders at the 24^th^ week used additional medications. Three of the nine patients who did not respond at 24^th^ week were receiving other medical treatments for their comorbidities. Of the 7 responders at 24^th^ week, only one received a multi-tablet regimen, whereas 4 out of 9 unresponsive patients at 24^th^ week were receiving multi-tablet therapy. Age, the number of comorbidities, and the use of multi-tablet regimens were higher in those who did not experience virological response at 24^th^ week.

In a study of Santoro et al. (4), 1,430 treatment-naive cases were divided into three groups according to viremia levels (≤30,000, 30,001–500,000, and >500,000 c/mL) and evaluated for virologic response to treatment. The lowest virological success rate at 48 weeks was observed in the group with viral load >500,000 c/mL. They stated that the revision of the frequently used 100,000 threshold value can be considered.

Sax et al. (5) wrote a letter on 41 treatment-naive cases with pretreatment virological load ≥1 million c/mL in phase 3 clinical trials. They found that the rate of virological success (HIV-RNA <50 c/mL) was 33% at 12^th^ week, 67% at 24^th^ week, and 97% at 48^th^ week. Similar to this study, our cases had lower virological response rates at 24^th^ week compared that at 48^th^ week.

Although the low number of patients in our study is the most important limitation, INI combination regimens were found to be very successful in patients with viral load >1 million c/mL. However, reasons such as comedicating or taking the drug in a single tablet or multiple tablets form may cause some disruptions in compliance and delays in virological response. In this context, we believe that the 24-week period for virological failure in the guidelines can be kept more flexible for these cases.

## Figures and Tables

**Figure 1:**
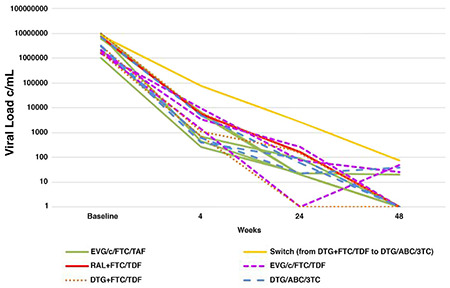
Virological response at 4^th^, 24^th^, and 48^th^ week. ABC: abacavir, DTG: dolutegravir, EVG: elvitegrevir, FTC: emtricitabine, RAL: raltegravir, TAF: tenofovir alafenamid, TDF: tenofovir disoproxil fumarate
